# Dual Wavelength Differential Detection of Fiber Bragg Grating Sensors with a Pulsed DFB Laser

**DOI:** 10.3390/s20174766

**Published:** 2020-08-24

**Authors:** François Ouellette, Zhonghua Ou, Jianfeng Li

**Affiliations:** State Key Laboratory of Electronic Thin Films and Integrated Devices, School of Optoelectronic Science and Engineering, University of Electronic Science and Technology of China (UESTC), Chengdu 610054, China; ozh@uestc.edu.cn (Z.O.); lijianfeng@uestc.edu.cn (J.L.)

**Keywords:** fiber Bragg grating, fiber optic sensor, dynamic chirp, DFB laser

## Abstract

We show how dual wavelength differential detection can be used to measure fiber Bragg grating sensors using nanosecond pulses from a single DFB laser diode, by taking advantage of its dynamic chirp. This can be performed in two ways: by measuring the reflected power from two separate pulses driven by two different currents, or by taking two delayed digitized samples within a single pulse. A prototype instrument using fast digitizing and processing with an FPGA is used to characterize the chirp, from which the performance can be optimized for both measurement schemes.

## 1. Introduction

Fiber Bragg grating (FBG) sensors have been used for three decades now [1], in applications as varied as aircraft wing shape measurements [2], temperature monitoring in medical treatments [3], pressure and temperature sensing in oil wells [4], or strain sensing in biomechanics [5]. FBG sensors have traditionally been measured either with a combination of a broadband optical source and a spectrometer, or a wavelength-swept source and a photodiode [1]. Both techniques involve acquiring a set of data points covering the entire range of wavelengths potentially reached by the sensor. The state of the sensor is determined by estimating the center of the Bragg grating spectrum from the resolution-limited spectral data, using more or less sophisticated algorithms [6,7]. Such spectral techniques allow the use of wavelength-division multiplexing (WDM) of multiple sensors along the same fiber, each sensor being an FBG with a different central wavelength. While FBG sensing systems have been used for many years, it is still felt that their more widespread use would benefit from more inexpensive, and higher resolution instrumentation.

Other interrogation techniques have nevertheless been proposed. For example, Morozov et al. [8] have proposed to probe the FBG with the two frequency sidebands of a modulated source. The state of the FBG can then be extracted from the amplitude and phase of the reflected modulated signal. Kulchin et al. [9] have proposed a scheme using an OTDR instrument, where the convolution of the reflected FBG spectrum with that of two or more reference FBGs can determine the state of the sensor. Those two techniques have some points in common with the technique presented here, such as the use of two frequencies, or the use of time-division multiplexing, though their implementation is quite different.

Recently, another technique to probe the state of an FBG sensor has been proposed and demonstrated [10,11,12,13,14,15,16,17]. It relies on measuring the reflectivity of the grating at two, or sometimes more, fixed wavelengths, all located within the spectrum of the FBG. The computed difference or ratio of the two or more reflectivity values can then be uniquely related to the location of the center of the spectrum. This technique has been dubbed dual-wavelength differential detection (DWDD) [13] and we shall use the same name and acronym here. Because it uses fixed wavelengths, DWDD lends itself more naturally to time-division multiplexing (TDM), where multiple identical sensors are interrogated by short optical pulses, with optical delays between the arrival times of each reflected pulse. Like other TDM schemes, this can be either in a serial configuration, with identical sensors distributed along one fiber, or in a parallel spatial-multiplexing configuration where the signal is split into multiple branches, each branch having a single sensor [18].

So far, most of the previous implementations of DWDD have have involved complex and expensive components and instruments. Wilson et al. [17] originally used a single laser diode, but relied on the diode operating on two longitudinal modes, one on each side of the grating spectrum, a condition not easy to obtain, and prone to mode partition noise. The two wavelengths also had to be separated by a monochromator before detection. Cheng and Xia [13] obtained a DWDD signal by taking the logarithmic ratio of the reflected signals from a tunable laser, manually switched between two wavelengths. To perform an equivalent TDM measurement, they used an electro–optic modulator to do a frequency sweep of the laser light, detecting it with an expensive network vector analyzer. Rohollanehjad et al. [16] used three tunable cw lasers, simultaneously modulated by an electro-optic modulator, and amplified by an EDFA, with three tunable filters to separate the wavelengths at detection. Xia et al. [14] used two pulsed tunable laser diodes with a programmable delay. Li et al. [15] measured both the static and dynamic strain and temperature on a chain of gratings, using a DWDD scheme for the static part. For this, they used two cw DFB laser diodes, amplified by switched semiconductor optical amplifiers, and further amplified by an EDFA, in order to avoid any effect of dynamic chirp. Apart from Wilson et al. [17], these demonstrations calculate the DWDD signal by subtracting the logarithms of the two signals, which does not eliminate noise due to power fluctuations of the source. Such a mathematically complex algorithm is also difficult to compute in real time for each pulse, and measurement rates in those demonstrations remains quite small.

We have recently demonstrated a much simpler and inexpensive DWDD instrument that uses a single, pulsed commercial DFB laser diode [11], and is suitable for TDM measurements of up to 15 sensors. We have also used a similar technique to measure tilted fiber Bragg gratings with very high resolution [12]. While other demonstrations have tried to avoid the dynamic chirp of the laser sources, we use the current and time dependence of the wavelength to our advantage in order to generate the two (or more) required wavelengths. In Reference [12], we have demonstrated that high signal-to-noise ratio detection and high resolution digitization, in conjunction with good wavelength stability of the source, or of an external reference, can achieve an effective spectral resolution better than 0.08 pm, which is a more than 10 times improvement over spectral instruments. These demonstrations show that DWDD instruments can offer an inexpensive, high performance alternative to spectral-based instruments for the interrogation of multiple FBG sensors.

**Figure 1:**
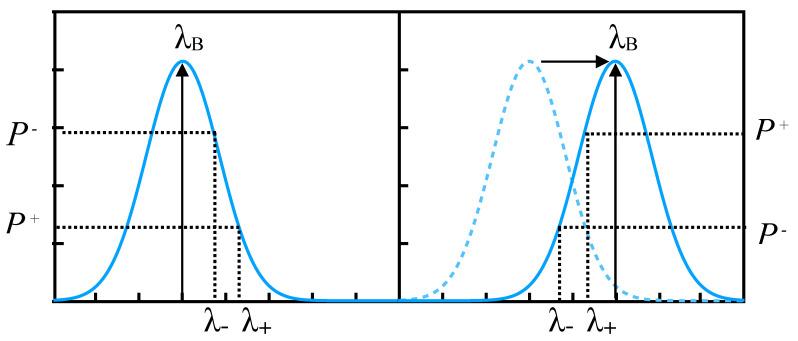
Dual-wavelength differential detection (DWDD) scheme involving the measurement of the reflected power at two closely spaced wavelengths $\lambda_{+}$ and $\lambda_{-}$. As $\lambda_{B}$ shifts, the difference between the two reflected powers also changes.

Our DWDD scheme is illustrated in Figure 1. We use measurements of the reflected power at two close wavelengths $\lambda_{+}$ and $\lambda_{-}$ separated by a fraction of the grating full-width at half-maximum (FWHM) $w_{B}$. As the Bragg wavelength $\lambda_{B}$ shifts under the influence of the measured parameter, the difference between the two reflected powers $P^{+}$ and $P^{-}$ changes. For example, as illustrated, as $\lambda_{B}$ shifts from $-w_{B}/2$ to $-w_{B}/2$ with respect to the average between those two wavelengths $\lambda_{av}$, the difference between the two reflected powers is inverted. It is thus essentially related to the slope of the spectrum at $\lambda_{av}$, and is a one-to-one function of $\lambda_{av}-\lambda_{B}$. Dividing the difference by the sum of the reflected powers normalizes the signal and makes it insensitive to the propagation loss between the source and the sensors, as well as to power fluctuations of the source. A Gaussian-shaped spectrum also results in a signal that is linear with respect to the Bragg wavelength, as was demonstrated in [10,12]. In [11], this simple algorithm was implemented in an FPGA chip, and could calculate the status of the sensor from nanosecond pulses in real time, leading to effective measurement speeds of more than 1 MHz for 15 simultaneously measured sensors.

We have previously studied in detail the dynamic chirp for a square-wave modulated DFB laser diode at a low frequency of 5 kHz, and how it could be used to measure tilted FBG sensors [12]. Here we investigate in more detail how knowledge of the dynamic chirp behaviour of a nanosecond-pulsed DFB laser diode can be used to maximize the sensitivity and resolution of the DWDD signal. Using a prototype instrument, we extract the relevant coefficients for the dynamic chirp, and demonstrate two methods by which the dynamic chirp can be used to get a DWDD signal: one using two pulses with different driving currents, and one using two digitized samples at different times within a single laser pulse.

## 2. DWDD Implementation with a Single DFB Laser

### 2.1. DWDD Algorithm and Resolution

If the wavelength of the DFB laser source is different at different times, then by digitizing the reflected signals, one can select the digitized samples that correspond to the desired probing wavelengths $\lambda_{+}$ and $\lambda_{-}$, and use them to compute the DWDD response. In our case, we use a simple algorithm involving basic arithmetic operations that can be implemented in an FPGA chip for real time calculation [10].

In order to estimate the resolution of our DWDD measurement, it should be noted that notions normally used for calculating the resolution of spectral scanning schemes, such as the bandwidth of the laser source, do not formally apply when one is dealing with chirped laser pulses. The wavelength of the laser is a time-varying quantity, and by measuring the average reflectivity over a given time interval, one is actually measuring the average reflectivity over a corresponding wavelength interval. For short enough time intervals, the reflectivity is a continuous function of the difference between the average wavelength and the FBG central wavelength. Since the DWDD scheme relies on calculating the difference in reflectivity between two such average wavelengths, the resolution is in fact related to the precision with which one can detect small changes in reflectivity.

We assume that the FBG spectrum has a Gaussian shape with a FWHM $w_{B}$. The Gaussian shape is a very good approximation for the central region of a weak uniform grating (R<30%). In cases where the emitted powers at the two wavelengths are different (as when we use pulses with different driving currents), the reflected signals $P^{+}$ and $P^{-}$ at wavelengths $\lambda_{+}$ and $\lambda_{-}$ can be normalized using a reference measurement of the pulse powers $P_{ref}^{\pm }$ as they are launched into the fiber. For example, we performed that by using the signal from the internal photodiode of the DFB laser. For cases where the power is the same at both wavelengths (as when we use two samples within the same square pulse), such normalization is not necessary. Considering the normalized powers $P_{n}^{\pm }=P^{\pm }/P_{ref}^{\pm }$, the following algorithm provides a sensing signal *S* that is linear with the Bragg wavelength [10,12]:

(1) $$S=\frac{P_{n}^{+}-P_{n}^{-}}{P_{n}^{+}+P_{n}^{-}}=\alpha \left(\lambda_{av}-\lambda_{B}\right),$$

where the sensitivity is given by the slope α:

(2) $$\alpha =4\ln\left(2\right)\frac{\delta }{w_{B}^{2}},$$

with $\delta =\lambda_{+}-\lambda_{-}$ being the wavelength separation.

The linear response is a result of the Gaussian shape of the spectrum and the small wavelength separation. An almost purely linear response ($r^{2}>0.998$) is obtained for $\delta \leq 0.4w_{B}$. The signal *S*, which is dimensionless, varies from positive to negative as $\lambda_{B}$ shifts from shorter wavelengths to longer ones. When $\left(\lambda_{av}-\lambda_{B}\right)$ becomes too large, the reflected powers become smaller, which increases the measurement noise. Therefore the measurement range is limited by the signal-to-noise ratio (SNR), and is proportional to $w_{B}$. Thus it can be tailored by changing the length, and therefore the bandwidth, of the grating, to match a particular sensing application. While we will show that the amount of chirp may place some limit on the bandwidth of the sensors, there are also ways to adapt the range using the packaging of the sensor itself, to make it more or less sensitive to a given parameter.

Because the range can be tailored in such a manner, and made to match the required range of the measured parameter, the performance of a DWDD instrument is best described by the ratio of the range to the resolution, which can be expressed as the number of bits of resolution $B_{r}$. This also makes it easier to compare with other sensing technologies. $B_{r}$ depends on the smallest measurable difference in the powers $P^{+}$ and $P^{-}$, which depends on the SNR and the resolution of the analog-to-digital converter (ADC). $B_{r}$ also depends on the slope α which governs how much change in power there is for a change in the Bragg wavelength. The resolution is largest when the average wavelength $\lambda_{av}$ coincides with the center of the grating spectrum at $\lambda_{B}$, and the reflected power is maximum. It then decreases as $\lambda_{B}$ moves away from $\lambda_{av}$. Considering a practical limit to the range as one FWHM $w_{B}$, the resolution at $\lambda_{av}-\lambda_{B}=0.5w_{B}$ can be expressed by [12]:

(3) $$B_{r}=B_{pd}-1-\log_{2}\left[1+\frac{1}{2\ln\left(2\right)\delta /w_{B}}\right].$$

$B_{pd}$ is the effective number of bits of resolution of the digitized and averaged photodiode signal as obtained for $\lambda_{av}-\lambda_{B}=0$, i.e., the maximum reflected power. This depends on both the effective resolution of the ADC, the SNR of the photodiode, and other sources of noise such as that from the laser current driver. Bits are also gained by averaging over *N* pulses, to the tune of $\log_{2}\sqrt{N}$. For nanosecond pulses, averaging can be performed over many thousands of pulses while still resulting in a measurement rate in the tens or hundreds of Hz, bringing significant resolution gain. For the case where the powers $P_{0}^{\pm }$ are not the same and a normalization to a reference pulse has to be done, $B_{pd}$ will be lower because it includes the noise from the reference measurements, and also because the lower of the two $P^{\pm }$ has a smaller SNR.

**Figure 2:**
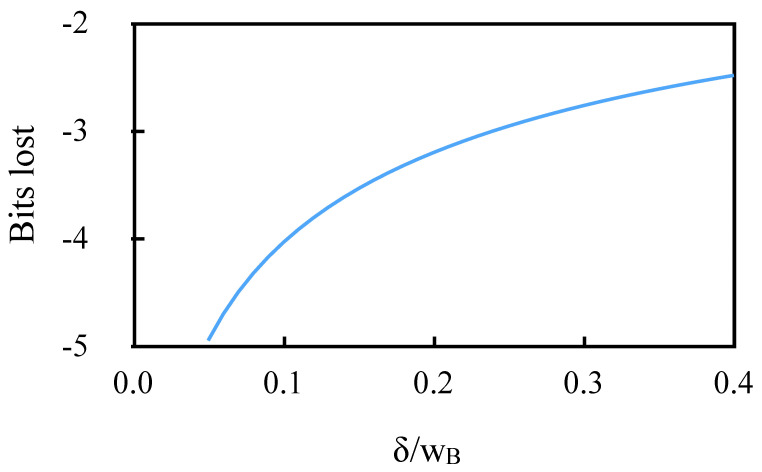
Resolution bits lost from $B_{pd}$ as a function of $\left(\delta /w_{B}\right)$.

One bit from $B_{pd}$ is then lost when the signal is at half the maximum possible reflected value. The last term in Equation (3) represents the effect of the sensitivity α, which depends on the ratio $\delta /w_{B}$. At a maximum practical value of $\delta /w_{B}=0.4$, this accounts for 1.5 more bits lost from $B_{pd}$. For smaller $\delta /w_{B}$, more bits are lost. Figure 2 shows the total bits lost from $B_{pd}$ as a function of $\delta /w_{B}$. Aside from maximizing the SNR, using a high value of δ is therefore essential to optimize the resolution of a DWDD instrument.

In Reference [12], we achieved nearly 12 bits of resolution, using a 14-bit ADC, with enough room for improvement to reach at least 13 bits, which would make the DWDD sensing system on par with competing technologies such as resistive strain gauges.

### 2.2. Dynamic Chirp of a Nanosecond-Pulsed DFB Laser

To generate two wavelengths with a significant wavelength spacing δ, we use the dynamic chirp of a single DFB laser diode, i.e., the dependence of its emission wavelength on both time and current. The wavelength of a DFB laser diode is directly related to the refractive index of its active region. When it is pulsed, two effects determine its behaviour. First, the injected current creates free carriers that change the refractive index and the wavelength in the negative direction. This change is linearly proportional to the current. On a time scale of nanoseconds, the concentration of free carriers follows the current adiabatically, thus this wavelength shift remains constant over the pulse duration for a square pulse. The second effect is the heating which occurs in the junction, which is also linearly proportional to the current, but has a more complex dynamic behaviour. The heat is generated in the active region, but gradually spreads to the entire diode chip, then its submount, and is ultimately dissipated by a heat sink in an active way if the temperature is controlled by a thermo-electric cooler (TEC). These different levels result in a temporal behaviour with as many as four different time constants, as has been shown by Shalom et al. [19]. Those time constants are all of different order of magnitude and range from about 15 ns to 50 μs. In our case, we use pulses of 5–30 ns, and therefore, following the same model as Shalom et al. [19], but keeping only the adiabatic effect and the nanosecond scale thermal effect, we can describe the chirp behaviour using the following equation:

(4) $$\lambda \left(t\right)-\lambda_{0}=I\left[A+B\left(1-e^{-t/\tau }\right)\right],$$

where t=0 is the beginning of the pulse, *I* is the current, τ is the thermal response time, and *A* and *B* are the adiabatic and thermal coefficients. $\lambda_{0}$ is the nominal resonant wavelength of the DFB laser cavity in the absence of current.

**Figure 3:**
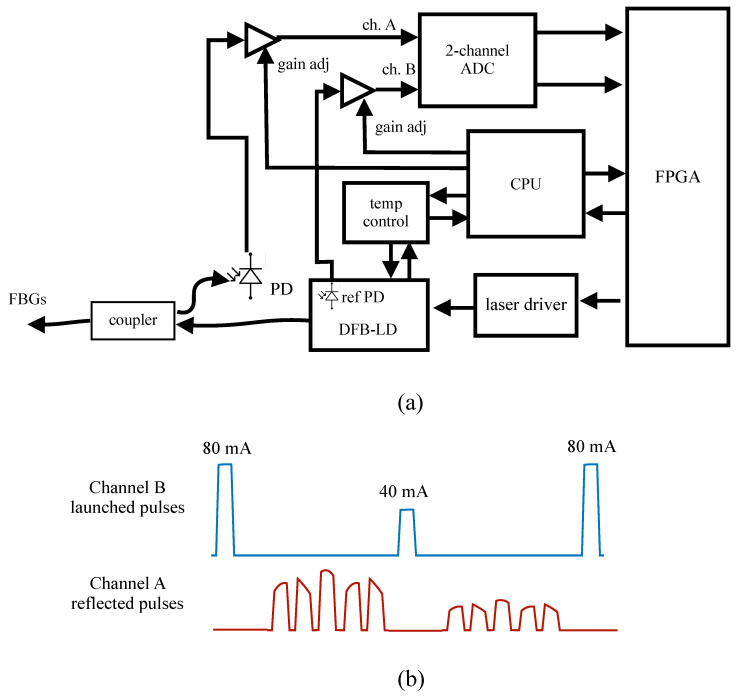
(**a**) Instrument schematic; (**b**) Temporal trace for the emitted pulses detected in channel B of the analog-to-digital converter (ADC), and the reflected pulses detected in channel A (in the case of 5 sensors).

**Figure 4:**
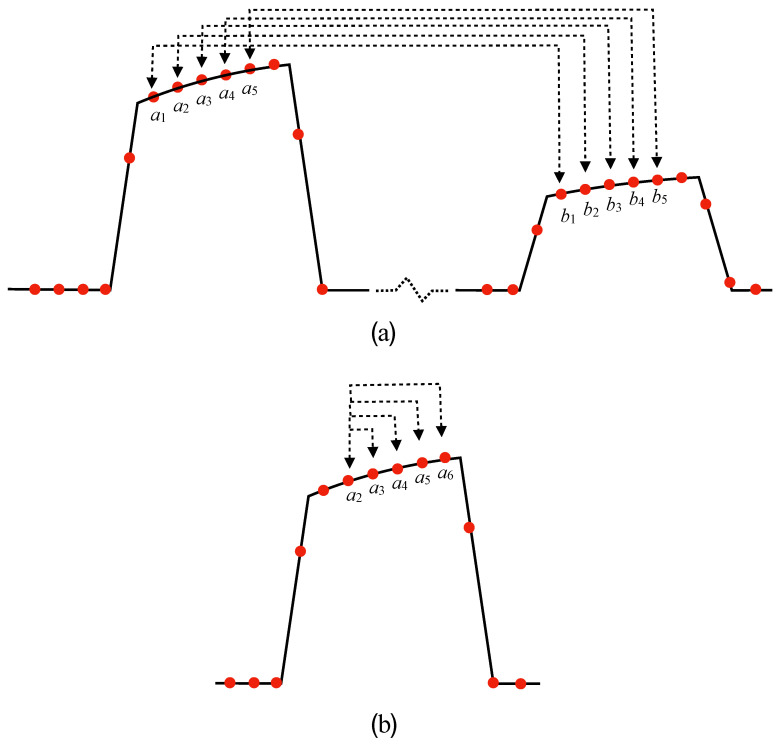
Digitized samples used for the measurements of *S* vs. the laser wavelength: (**a**) two-pulse scheme and (**b**) one-pulse scheme.

Since the dynamic chirp is linearly proportional to the current, one way to exploit it is to use two pulses emitted in succession, with different driving currents, and therefore two different wavelengths. We call this scheme the two-pulse scheme. Enough delay is allowed between the pulses for the reflections from all the sensors to reach the photodiode, as illustrated in Figure 3b. On the other hand, since the wavelength also evolves along a single pulse, one can also use two digitized samples at different times along the pulse, as was done in Reference [12] for a square-wave modulated laser diode. We call this the one-pulse scheme, which is illustrated in Figure 4b. The achievable value of δ for both schemes requires a knowledge of the coefficients in Equation (4).

**Figure 5:**
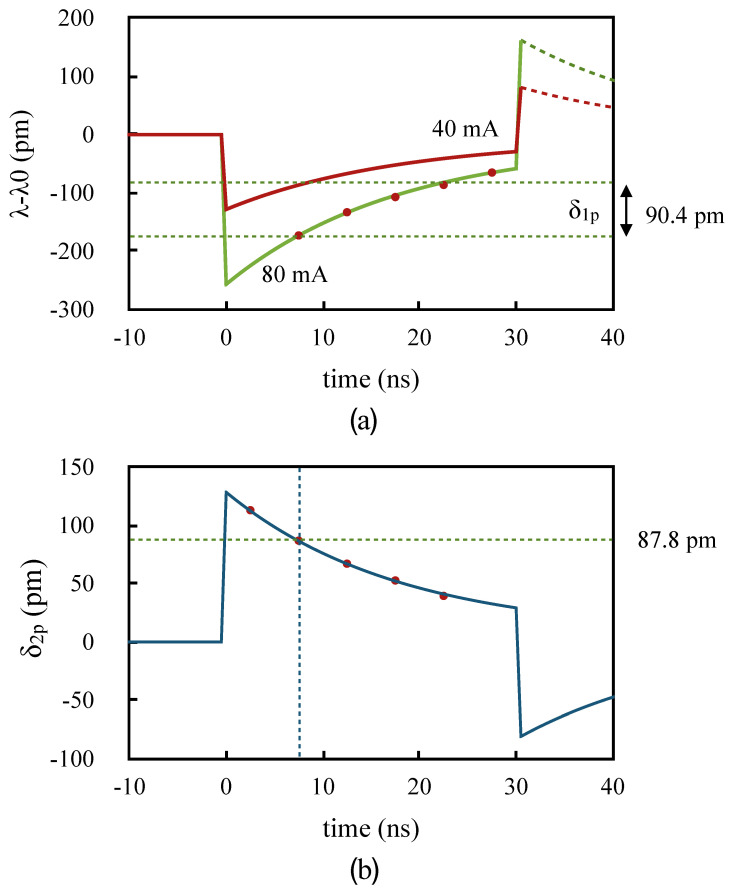
(**a**) Wavelength vs. time for 80 mA and 40 mA, 30 ns pulses; (**b**) Difference $\delta_{2p}$ between the wavelengths of the 80 mA and 40 mA pulses. The dots are experimental measurements. The dotted lines in (**a**) represent the expected value of $\delta_{1p}$ at 7.5 ns, and in (**b**) that of $\delta_{2p}$ for 15 ns delay from the 7.5 ns sample.

In our prototype instrument, we chose to use pulses with 80 mA and 40 mA driving currents. Such a choice was made to have a large enough value of δ, while not sacrificing too much SNR in the second pulse. Figure 5a illustrates the temporal behaviour of the emission wavelength according to the model of Equation (4), with respect to the nominal wavelength $\lambda_{0}$, for two perfectly square 30 ns pulses with these two driving currents, the wavelength shift for 40 mA being half that for 80 mA. Figure 5b shows the time dependence of the difference $\delta_{2p}$ between the wavelength of the pulses for the two-pulse scheme. The values of *A*, *B*, and τ used in Figure 5 are those found in our experiments described below, and the dots are the experimental measurements. In practice, the pulse rise and fall times were about 1 ns, which does not affect the model significantly.

According to the model of Equation (4), as the current rapidly rises, the wavelength drops simultaneously. Following this fast drop, the wavelength then rises with an experimentally determined time constant of 17.8 ns. After the end of the pulse, the wavelength rises rapidly because the adiabatic contribution vanishes, and then decreases with the same thermal time constant as the laser junction cools down. This is however not observable because there is no more optical power being emitted, hence it is shown as a dashed line in Figure 5a.

In practice, there is also a slower rise due to the thermalization processes on longer time scales, but that is not significant on the nanosecond time scale. This slower thermal effect gives rise to a steady state wavelength shift, which depends on the average driving current and the repetition rate of the pulses. However, we do not need to consider it here.

The relative contributions of the adiabatic and thermal shifts were found to have nearly equal magnitude, though of opposite signs, consistent with the values measured by Shalom et al. [19]. Because of this, we found that the one-pulse and two-pulse schemes can have an equivalent sensitivity, given enough delay between the samples of the one-pulse scheme.

For the two-pulse scheme, the wavelength difference $\delta_{2p}$, and therefore the sensitivity, is higher at the beginning of the pulses, as can be deduced from Figure 5b. Therefore, maximum sensitivity is obtained for shorter pulses. For the one-pulse scheme, we found that the wavelength difference $\delta_{1p}$ when using samples at *t* and t+15 ns, is nearly the same as $\delta_{2p}$ obtained using the samples at time *t* in the 40 mA and 80 mA pulses. This is illustrated by the dashed lines in Figure 5b, which show the value of $\delta_{2p}$ for a sample taken 7.5 ns from the start of the pulse (87.8 pm), and in Figure 5a, which show the value of $\delta_{1p}$ from the difference in wavelength between a sample taken 15 ns later than the 7.5 ns sample (90.4 pm).

In the following sections, we describe the design of our prototype instrument, and the measurements made to obtain the data, and extract the parameters used in Figure 5.

## 3. Instrument Design

Our prototype instrument was originally conceived and designed with the two-pulse measurement scheme in mind. As the laser source, we use a commercial DFB laser diode (Jiuzhou EO) mounted in a butterfly package with internal TEC, isolator, and monitor photodiode. It has a rated power of 8 mW for a 90 mA current. The wavelength of the DFB laser diode is also a function of temperature. This feature is useful for fine-tuning the laser to the centre of the desired sensing range. From the spectrum of the laser diode at various temperatures, we obtained a linear response of 97 pm/${}^{\circ }$C over a 10 ${}^{\circ }$C range, in line with the laser specifications.

In order to implement a TDM scheme with sufficiently short distances between the gratings, pulses in the nanosecond range are required, and the processing circuitry must be accordingly fast. We chose a low cost 10 bit, dual-channel ADC, with 200 Msps per channel (TI ADC10DV200). Thus the signal is sampled in 5 ns intervals. One channel was used to measure the reflected pulses, while the other one measured the reference pulse from the laser diode internal monitor photodiode. The sampled signals are then fed to the FPGA (Xilinx Spartan 6 LX25) for fast calculations of the DWDD signals. Both the laser driver (Maxim 3967AETG+) and the detection and amplification circuits have a response time of about 1 ns.

The instrument schematic is shown in Figure 3a, while Figure 3b illustrates (not actual data) the sequence of lauched pulses detected in channel B of the ADC and the received pulses from the multiple FBGs detected in channel A. A first pulse at 80 mA was launched, and time was allowed for the echoes from up to 15 sensors to be received by the photodiode and digitized by the ADC (though only 5 are shown in the Figure). A second pulse at 40 mA was then launched, and the echoes were similarly detected and digitized. The FPGA then performed calculation of the 15 DWDD signals in real time, after which another pair of pulses is launched. All the components were mounted on a PCB (designed and fabricated by IPCB Systems, Bromont, QC, Canada). A CPU controls the various components, and communicates with a computer via a USB link, where the acquisition parameters can be selected from a user interface.

The user interface also has an oscilloscope mode, where the values of the signals for each sample along the acquisition sequence can be visualized graphically, showing the reflected pulses like an oscilloscope trace. They can also be measured directly by zooming in on the trace, with digitized values from 0 to 512 for positive signals. We used this mode to directly measure the reflected powers in order to calculate the value of *S* for the one-pulse scheme, without having to reprogram the FPGA.

The instrument was designed with a target ratio of range to resolution of at least 1000. Since *S* is not expected to be larger than 1, only four significant decimal digits are displayed. In the rest of the paper, we will display the *S* values multiplied by 10,000, so that they are represented by a dimensionless integer corresponding to those four significant digits.

**Figure 6:**
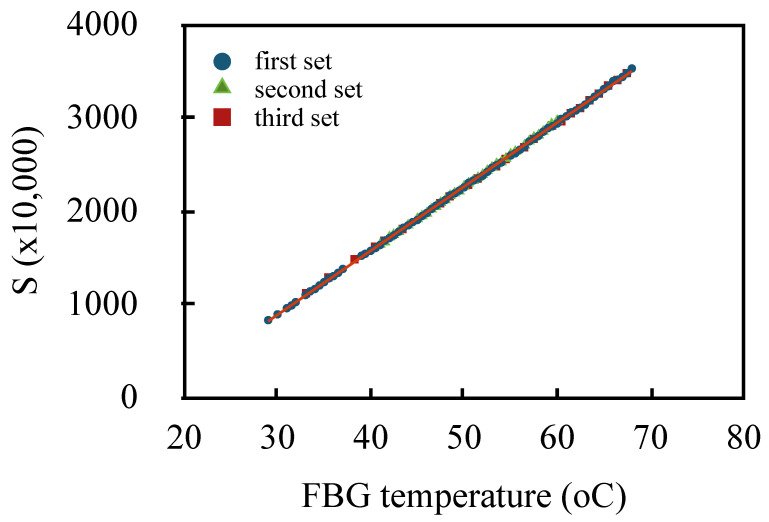
Signal *S* as a function of the FBG sensor temperature for three measurement sets.

This instrument showed high performance for measuring FBGs as temperature sensors [11]. As an illustration, Figure 6 shows three sets of measurements of *S* as a function of temperature for FBGs immersed in water, using the two-pulse scheme with 5 ns pulses. The FBG had 710 pm bandwidth and about 1.5% reflectivity. Over the temperature range, the signal had extremely good linearity ($r^{2}>0.9996$) and the measurements were highly repeatable. Taking into account the noise in the value of *S*, we estimated the effective resolution as 0.05 ${}^{\circ }$C. More details on the instrument performance in measuring multiple sensors will be given elsewhere.

## 4. Characterization of the Dynamic Chirp

**Figure 7:**
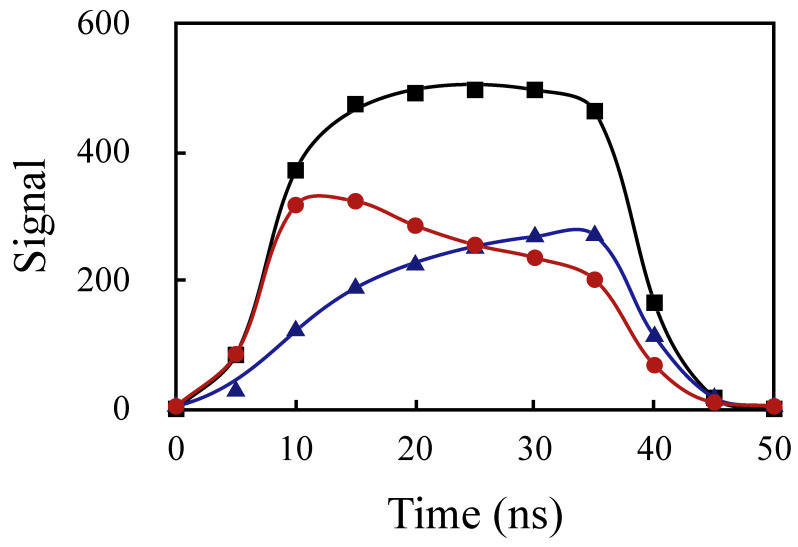
Pulse shape after reflection for three laser diode temperatures corresponding to the center of the grating (squares), and at about half-maximum on both sides (circles and triangles).

The effect of the dynamic chirp could be readily visualized from the reconstructed pulse shapes in the oscilloscope mode of our instrument, as can be seen in Figure 7. When the laser diode average wavelength is on the side of the FBG spectrum (at about half-maximum), the reflection increases or decreases with time, depending on which side. When the average wavelength is centered on the Bragg wavelength, the reflection is flat across the pulse. This strong reshaping indicates that the amount of chirp is a significant fraction of $w_{B}$.

In order to characterize the dynamic chirp with more precision, we measured the value of the slope α of the signal *S* as a function of the laser wavelength when using different pairs of digitized samples, either between the 80 mA and 40 mA pulses, or within the 80 mA pulse, as shown in Figure 4. Instead of scanning the grating temperature, which can be time consuming, we performed the measurements by scanning the laser diode temperature, knowing that it shifts with a value of 97 pm/${}^{\circ }$C. The slope of the resulting curves was then used to calculate the value of the wavelength difference δ from Equation (2). $w_{B}$ had previously been obtained by recording the reflected power as a function of the laser diode wavelength, and fitting it to a Gaussian with $w_{B}=710$ pm.

**Figure 8:**
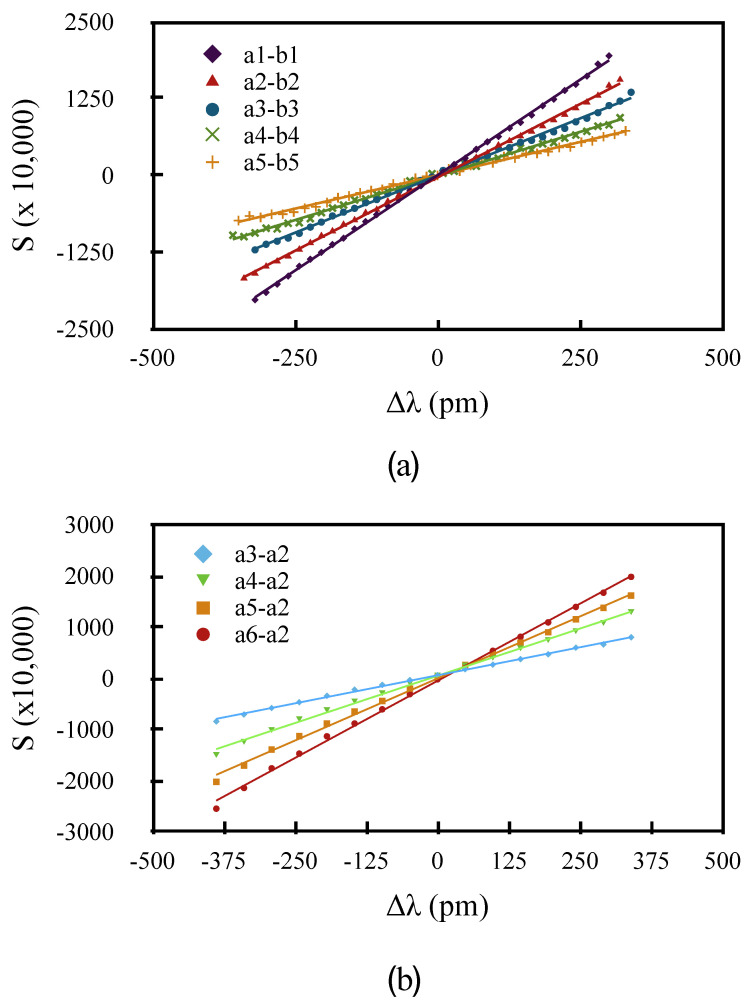
S vs. Δλ for (**a**) two-pulse scheme, and (**b**) one-pulse scheme illustrated in Figure 4.

Denoting the samples in the 80 mA pulse as $a_{n}$ and in the 40 mA pulse as $b_{n}$, in the first set of measurements, illustrated in Figure 4a, we used 30 ns pulses and measured *S* using five corresponding pairs of samples $a_{1}-b_{1}$ to $a_{5}-b_{5}$, starting from about 2.5 ns into the pulse. All the linear fits had a regression coefficient $r^{2}>0.99$, and thus gave an accurate value of δ. The five curves are shown in Figure 8a. According to Equation (4), and for a difference in driving currents of 40 mA, the values of $\delta_{2p}$ obtained from the slopes should follow:

(5) $$\delta_{2p}\left(t\right)=\lambda_{+}\left(t\right)-\lambda_{-}\left(t\right)=40\left[A+B\left(1-e^{-t/\tau }\right)\right].$$

A second set of measurements was made by calculating *S* from the reflected power for the 80 mA pulse, using pairs of sample $a_{2}-a_{3}$, $a_{2}-a_{4}$, $a_{2}-a_{5}$ and $a_{2}-a_{6}$, thus effectively measuring $\lambda_{+}\left(t\right)-\lambda_{+}\left(t_{0}\right)$ along the pulse relative to the second sample at $t=t_{2}$. Those curves are shown in Figure 8b. In this case, the measured values $\delta_{1p}\left(t\right)$ should be given by:

(6) $$\delta_{1p}\left(t\right)=\lambda_{+}\left(t\right)-\lambda_{+}\left(0\right)=80B\left(1-e^{-t/\tau }\right).$$

From Equations (5) and (6), we find that:

(7) $$\delta_{2p}\left(t\right)-\delta_{2p}\left(0\right)=\delta_{1p}\left(t\right)/2=40B\left(1-e^{-t/\tau }\right)$$

**Figure 9:**
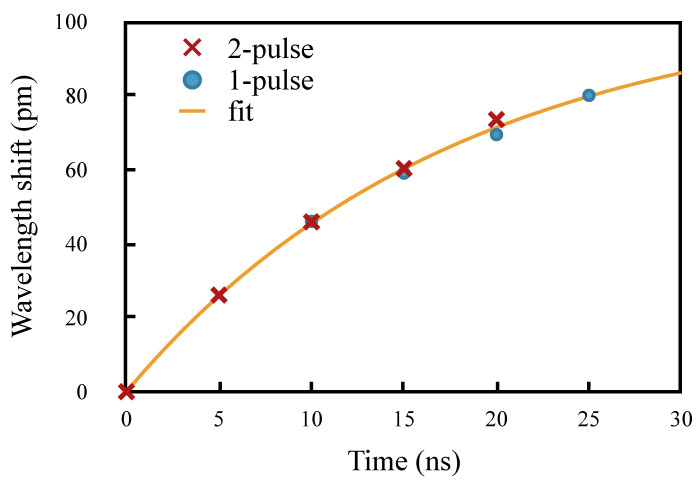
Best fit for *B* and τ.

Therefore the two sets of measaurements can be combined to fit Equation (7), which is shown in Figure 9, from which we determined the values of *B* and τ. Using these values, *A* could then be estimated from the $\delta_{2p}\left(t\right)$, using:

(8) $$A=\frac{\delta_{2p}\left(t\right)}{40}-B\left(1-e^{-t/\tau }\right).$$

The best estimate for *A* was obtained by averaging the results of Equation (8) from the five experimental values of $\delta_{2p}$. The curves of Figure 5, which include the data points for both sets of measurements, were plotted using the parameters from this fit. The values obtained were *A* = −2.81 pm/mA, *B* = 2.64 pm/ma, and τ = 17.8 ns. These were within the range of those obtained by Shalom et al. [19] for different models of DFB lasers.

With these parameter values in hand, we can now compare the relative sensitivities of the one-pulse and two-pulse schemes, and find how much delay between the samples of the one-pulse scheme gives an equal sensitivity to the two-pulse scheme. Given the time $t_{0}$ of the first sample, taken from the onset of the pulse, the delay between samples Δt for equal sensitivity of both schemes is:

(9) $$\Delta t=-\tau \ln\left[\frac{1+\left(1+\frac{A}{B}\right)\;e^{t_{0}/\tau }}{2}\right].$$

**Figure 10:**
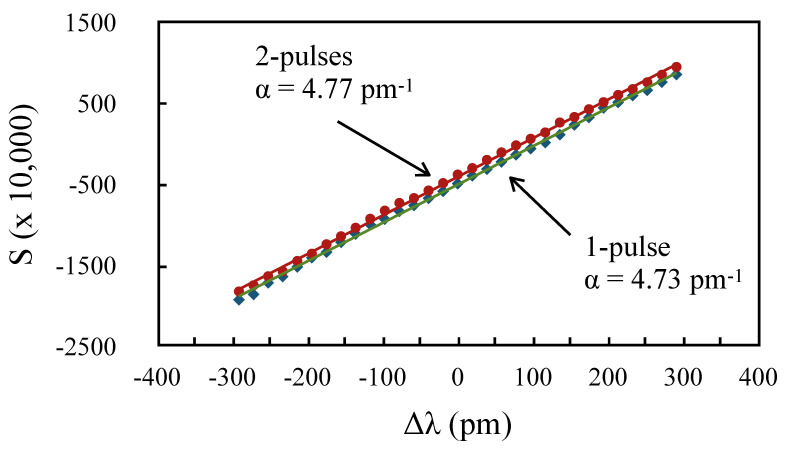
*S* vs. Δλ for both schemes, for 15 ns delay between samples in the one-pulse scheme.

Because A≈−B, the dependence on $t_{0}$ was small, and Δt varied from 13.5 ns to 14.1 ns for $t_{0}$ going from 0 to 8 ns. Therefore, a three-sample delay (15 ns) should give about the same sensitivity. To confirm this, we performed one more set of measurements of *S* using the samples $a_{2}-b_{2}$ of a 40 ns pulse in the two-pulse scheme, and the samples $a_{2}-a_{5}$ for the one-pulse scheme, as shown in Figure 10. An essentially similar slope was obtained for both schemes. For larger Δt, however, the sensitivity of the one-pulse scheme would exceed that of the two-pulse scheme. Whereas, the value of $\delta_{2p}$ is limited by $(I_{+}-I_{-})A=112$ pm, that of $\delta_{1p}$ is limited by $I_{+}B=211$ pm. For example, for a 35 ns delay, the one-pulse scheme is 60% more sensitive than the two-pulse scheme.

## 5. Discussion and Conclusions

The use of a pair of short pulses has the advantage that a shorter delay between the pulses can be used. For sensors along a single fiber, this means a shorter spacing between the sensors. In a parallel configuration, less fiber is required to impart a delay between each branch. Using 5 ns pulses with 5 ns latency between pulses means that a 1 m spacing can be used.

On the other hand, the one-pulse scheme has many advantages of its own. First of all, for applications that do not require such short spacing between the sensors, the one-pulse scheme can have a larger sensitivity for longer delays between samples. Secondly, for a square pulse, the power is essentially constant during the pulse. Therefore, there is no need to normalize to a reference power. The algorithm for *S* is then even simpler and faster to compute. Since it involves fewer measured quantities, the compound error is also smaller. Finally, the single pulse can use the maximum driving current allowed by the laser diode, which maximizes the power and thus the SNR. As mentioned in Section 2, the resolution as given by Equation (2) will be smaller when a power normalization is performed, as in the two-pulse scheme. Though the exact number of bits lost depends on the details of the instrument, it should represent at least one bit in a typical case.

In terms of measurement rate, for an equivalent sensitivity, both schemes are pretty much on par. Given a latency time of 5 ns between each pulse, to account for the finite response time of the detection system, the two-pulse scheme requires two such periods for each sensor. Thus the total measurement time for each sensor is twice the sum of the pulse duration plus the latency time, which is 20 ns. The one-pulse scheme requires 20 ns for the pulse, to ensure that the 2 probed samples separated by 15 ns are within the pulse, and 5 ns latency time, for a total of 25 ns. For 15 sensors, this represents basic measurement rates of 3.3 and 2.7 MHz. No application requires such a fast measurement rate, and a faster rate is really only useful because more averaging can be performed. The 30% difference would only provide a fraction of improvement in the final resolution.

One important aspect of the use of DWDD as a commercial system is that it allows, and actually requires, both the sensors and the instruments to be standardized. All instruments, and all sensors, should function around the same standardized wavelength. While this standardization requirement can be seen as a limitation, because it imposes stringent requirements on reproducibility of the components and manufacturing process, it can also be seen as an enabler for widespread deployment. For example, similar standardization has enabled the wide deployment of dense wavelength division multiplexing (DWDM) systems. The DFB laser diode used for this work indeed has a precise wavelength because it is intended for use in such systems. Precisely wavelength-matched FBG filters for DWDM systems are also commonly available. Standardization reduces costs, because it allows components to be mass produced without modifying the production line, and to be stocked in inventory for rapid, on demand delivery. By contrast, current WDM-based FBG sensor systems are practically all custom-designed and custom-made, which makes them economically viable only for niche applications.

As a related point, it is well-known that the characteristics of DFB lasers (central wavelength, chirp) can age and drift over time, and also that individual devices will have different characteristics. Therefore, a DWDD instrument, like any other sensing instrument, requires calibration to ensure accurate measurement, as should the sensors used with it. This is typically done using so-called golden units, that are well-calibrated sensors with properties that can ultimately be traced to national or international standards. This is actually a much more subtle and complicated issue than it might seem at first sight, and is most often avoided in much of the FBG sensor literature. In the case of DWDD, we expect to address it more fully in our future work. As for ensuring long-term accuracy, we previously discussed [12] how a sensible way of ensuring it is to use FBGs themselves as internal references. Properly annealed FBGs are known to have a great degree of long-term stability. A pair of properly-positioned reference FBGs can account not just for the wavelength drift, but also for the change in calibration slope brought about by potential change in the chirp coefficient δ. In a TDM-based instrument, those reference FBGs only take two slots in the measurement sequence, still leaving multiple slots for the actual sensors. Furthermore, the temperature dependence of the FBG wavelength is one tenth that of a typical DFB laser, so temperature-controlled reference FBGs can have extremely high wavelength stability. That way, a DWDD instrument can achieve a resolution unattainable with spectral scanning instruments [12].

In conclusion, we have shown that the dynamic chirp of a single DFB laser diode can be used to perform dual-wavelength differential detection of fiber Bragg grating sensors with nanosecond duration pulses, using a simple algorithm that can be implemented in a fast FPGA chip for real time calculation. Because the adiabatic and thermal contributions to the dynamic chirp have opposite sign but nearly equal magnitude, we have shown that the DWDD measurement can be implemented in two ways with equivalent sensitivity: either use two 5 ns pulses with different driving currents, or use two samples within a single pulse, separated by at least 15 ns.

Because of its simplicity and low cost, the DWDD method implemented in this way opens the way to inexpensive, high-resolution FBG sensing systems, making them competitive with other sensing technologies in both price and performance.

## Abbreviations

The following abbreviations are used in this manuscript:

DWDD	Dual wavelength differential detection
DFB	Distributed feedback
FBG	Fiber Bragg grating
FPGA	Field programmable gate array
TDM	Time division multiplexing
EDFA	Erbium doped fiber amplifier
FWHM	Full width at half maximum
SNR	Signal to nois ratio
ADC	Analog to digital converter
TEC	Thermoelectric cooler
PCB	Printed circuit board
CPU	Central processing unit
DWDM	Dense wavelength division multiplexing
